# The Association of Central Corneal Thickness and Intraocular Pressure Measures by Non-Contact Tonometry and Goldmann Applanation Tonometry among Glaucoma Patients

**DOI:** 10.4314/ejhs.v30i6.18

**Published:** 2020-11

**Authors:** Samuel Kyei, Frank Assiamah, Michael Agyemang Kwarteng, Cynthia Pakyennu Gboglu

**Affiliations:** 1 Department of Optometry and Vision Science, School of Allied Health Sciences, College of Health and Allied Sciences, University of Cape Coast, Cape Coast, Ghana; 2 Department of Optometry, Faculty of Science and Engineering, Bindura University of Science Education, Bindura, Zimbabwe; 3 Discipline of Optometry, College of Health Sciences, University of KwaZulu-Natal, South Africa

**Keywords:** Central corneal thickness, Goldmann Applanation Tonometer, Non-contact Tonometer, Intraocular Pressure, Ghana

## Abstract

**Background:**

The aim of this study was to determine whether Central Corneal Thickness (CCT) is associated with intraocular pressure measurement (IOP) with a Non-contact tonometer and the Goldmann applanation tonometer in glaucoma patients.

**Materials and Methods:**

The study involved two hundred and thirty-two eyes of clinically diagnosed glaucoma patients receiving care at a referral facility. IOP measurements were obtained using both the Non-Contact Tonometer (NCT) and Goldmann Applanation Tonometer (GAT). The association between age, ethnicity, and CCT, as well as CCT on the measures of NCT and GAT, were analyzed.

**Results:**

There were 64(55.2%) males and 52 (44.8%) females and their ages ranged from 18 to 85 years (mean age = 47.72; SD ± 15.75 years). There was a weak positive correlation between the CCT and NCT findings in the right eye (r = 0.19, n = 116, p < 0.05) and in the left eye (r = 0.25, n = 116, p < 0.05). However, there was no correlation between CCT and GAT measurements. Age had a significant correlation with CCT while gender and ethnicity had no significant correlation.

**Conclusion:**

The study found a significant association between CCT and NCT. However, there was no significant association between CCT and GAT. CCT had an association with age but independent of gender and ethnicity since there was no significant relationship between these variables.

## Introduction

Central Corneal Thickness (CCT) has become an important parameter to consider in the diagnosis of glaucoma (1). The development and progression of glaucomatous damage are facilitated by elevated intraocular pressure (2). This warrants an accurate intraocular pressure (IOP) assessment which impacts treatment modalities in patients presenting with glaucoma (2). Intraocular pressure remains the main modifiable risk factor of glaucoma through therapeutic and surgical modalities (3).

Over the decades, Goldmann Applanation Tonometer (GAT), Non-Contact Tonometer (NCT) among others have been developed in the assessment of intraocular pressure (4,5). Several studies have been conducted to compare IOP measurements using GAT and NCT, and the effect of CCT on these measurements (6–9). CCT has been found to decrease with age, and gender has been found to be associated with CCT (10). It has been reported that the average CCT is 530–540 µm, with a variation of 60 µm or 70 µm among healthy eyes (10). Variations in CCT have implications for intraocular pressure measures and refractive status (10–12).

There is a need to consider the composite effect of age, gender, ethnicity, and central corneal thickness on IOP measurements using NCT and GAT among a sample of glaucomatous patients in Ghana. This will inform practitioners and researchers on the modalities to consider in the measurement of intraocular pressure among Ghanaians with glaucoma.

## Materials and Methods

**Study setting**: This study was carried out at the premises of the Bishop Ackon Memorial Christian Eye Center, Cape Coast. The center is the most utilized Christian eye care facility in the Cape Coast metropolis of Ghana.

**Study design**: This was a clinic-based prospective study of patients with glaucoma visiting the eye clinic from January 2019 – December 2019. The study involved the comparison between Goldman Applanation Tonometric (GAT) measures, Non-Contact Tonometric (NCT) measures and pachymetry readings of central corneal thickness. The age and ethnic background of participants were also recorded.

**Sampling technique**: The sampling method was non-probability convenience sampling. The sampling method was based on the fact that the study involved all clients with glaucoma visiting the clinic during the study period.

**Inclusion and exclusion criteria**: The study included all clients with glaucoma who were 18 years and older. The study excluded clients with any pre-existing ocular surface disease, corneal disease, eye surgery, ocular trauma and inflammatory eye diseases.

**Ethical considerations**: The study adhered to the tenets of the Declaration of Helsinki, and approval was sought from the Institutional Review Board of the University of Cape Coast (UCCIRB/CHAS/2019/178). The informed consent of the participants was obtained. The procedure was explained to the participants and the risk of minimal discomfort involving the contact of the Goldmann tonometer probe with the cornea. They were assured the anesthesia will help in the relief of the discomfort. No financial remunerations were offered to the participants. Participation in this study was voluntary, and participants were informed that they could withdraw their participation at any point and that in the event of refusal/withdrawal of participation, there would be penalty or loss of treatment or other benefits to which they would normally be entitled.

**Data collection procedure**: Data collection involved collation of data on demographics, CCT and IOP measures in the following manner:

1. The examination of the anterior segment was performed on each participant using a slitlamp biomicroscope.
2. The examination of the posterior segment was conducted with an ophthalmoscope.
3. Central corneal thickness was measured using Wavelight oculyzer II (Alcon Surgical, Fort Worth, Texas, USA).
4. Intraocular pressures were measured using the Slit lamp mounted Goldmann AT 900 (Haag Strait, Bern, Switzerland) and a Topcon CT80 Non-Contact Tonometer (Topcon Medical, NJ, USA) on each participant. The NCT followed by GAT measurement was done in triplicate on each eye at interval of 5 minutes recovery time between each measurement. The method was started with NCT then GAT at an interval of 10 minutes. ALCAINE® 0.5% eye drops (Alcon Laboratories Inc., Fort Worth, TX, USA) were instilled on the eyes for anesthesia, and fluorescein strips (Medical Equipment India, Kabir Nagar, New Delhi, Delhi) were used for staining of the cornea.
5. All measurements were taken by a single experienced optometrist.

**Data analysis**: Data were analyzed using the IBM SPSS version 21 (SPSS Inc, Chicago, USA). Categorical data were presented as frequencies. Pearson correlation coefficient was used to determine the association between CCT and GAT, NCT, age, gender, and ethnicity. P<0.05 was considered statistically significant.

## Results

**Demographics**: A total of 232 eyes of 116 participants were involved in the study. There were 64(55.2%) males and 52(44.8%) females, and their age ranged from 18 to 85 years (mean age = 47.72; SD ± 15.75 years) (Tables 1 and 2).

**Table 1:** Distribution of Age according to gender

Age group	Gender		Total
	Male	Female	
**Youth (18–35)**	18	9	27
**Adult (36–59)**	30	33	63
**Elderly (>60)**	16	10	26

**Total**	64	52	116

**Table 2:** Distribution of ethnicity according to gender

Ethnicity	GENDER		Total
		
	Male	Female	
**Akan**	42	31	73
**Guan**	7	8	15
**Ewe**	5	6	11
**Ga Adangbe**	10	7	17

**Total**	33	27	116

**Goldmann Tonometric, Non-Contact Tonometric, and Central Corneal Thickness Readings**: The median GAT measurement in the right eye was 17.0 mmHg, and that of the left eye was 16.0 mmHg. The median NCT measurement in the right was 18.0 mmHg, and in the left eye was 17.0 mmHg. The median CCT was 537.0 *µ*m and 534.0 *µ*m in the right and left eyes respectively (Table 3).

**Table 3:** Means of CCT, GAT, and NCT

Variables	Mean ± SD	95% CI	Median	Range
**OD CCT**(*µ*m)	532.1 ± 27.9	526.9 – 537.3	533.0	465.0 – 592.0
**OS CCT**(*µ*m)	531.1 ± 28.0	525.9 – 536.3	534.0	465.0 – 593.0
**OD GAT**(mmHg)	18.33 ± 8.44	17.15 – 21.51	17.00	10.00 – 58.00
**OS GAT**(mmHg)	18.13 ± 9.80	16.60 – 21.67	16.00	10.00 – 56.00
**OD NCT**(mmHg)	19.30 ± 6.87	18.04 – 20.56	18.00	12.00 – 58.00
**OS NCT** (mmHg)	19.65 ± 9.13	17.97 – 21.33	17.00	12.00 – 57.00

OD-Oculus Dexter, OS-Oculus Sinister, NCT-Non-Contact Tonometry, GAT-Goldmann Aplannation Tonometry, SD-Standard deviation, CI-Confidence interval

**Correlations between central corneal thickness (CCT), GAT, and NCT**: Pearson product-moment correlation coefficient was computed to assess the relationship between CCT, GAT and NCT. There was a weak positive correlation coefficient between CCT readings in the right eye and NCT in the right eye (r = 0.19, n = 116, p < 0.05). Also, there was a weak positive correlation coefficient between CCT readings in the left eye and NCT in the left eye (r = 0.25, n = 116, p < 0.05). The correlations between CCT, GAT and NCT readings are shown in Table 4. Also, controlling for age as a confounding factor, a partial correlation analysis between CCT, gender, GAT and NCT readings were computed (Table 4).

**Table 4:** Correlation between CCT and GAT, NCT, Age

Variables	R	R^2^	*P*-value
**OD CCT VS OD GAT**	0.09	0.01	0.23
OD CCT VS OD GAT*	0.10	-	0.17
**OS CCT VS OS GAT**	0.06	0.003	0.45
OS CCT VS OS GAT*	0.08	-	0.29
**OD CCT VS OD NCT**	0.19	0.04	0.04
OD CCT VS OD NCT*	0.22	-	0.02
**OS CCT VS OS NCT**	0.25	0.06	0.01
OS CCT VS OS NCT*	0.29	-	0.002
**OD CCT VS Age**	-0.22	0.78	0.02
**OS CCT VS Age**	-0.22	0.85	0.02
**OD CCT VS Gender**	0.04	-	0.68
OD CCT VS Gender*	0.06	-	0.52
**OS CCT VS Gender**	-0.04	-	0.68
OS CCT VS Gender*	-0.02	-	0.84

OD-Oculus Dexter, OS-Oculus Sinister, NCT-Non-Contact Tonometry, GAT-Goldmann Applanation Tonometry, CCT-Central Corneal Thickness

* Partial correlation when age is controlled

## Discussion

This study determined the effect of CCT on the IOP measures of GAT and NCT in glaucoma patients in Ghana. The participants were mainly adults with a mean central corneal thickness (CCT) of 532.1*µ*m and 531.1*µ*m in the right and left eyes respectively. This finding is similar to a study by Ntim-Amponsah *et al.* (13), who reported a mean CCT of 533.3 *µ*m among a healthy Ghanaian population with a mean age of 34.09 ± 12.14. An average CCT of 530–540 *µ*m has been reported as the normative value among healthy adults (10). The mean CCT obtained in this study was within normal CCT measures.

Central corneal thickness plays an important role in the assessment of glaucoma. Studies have reported that a thinner CCT is a predictive factor for the progression of primary-open angle glaucoma (14–16). The predictive role of CCT for glaucoma prognosis was not within the scope of this study since participants were already patients with glaucoma and receiving care. Nonetheless, the minimum CCT (465*µ*m) (Table 3) observed in both eyes signifies advanced glaucomatous damage (17–19).

This study found a significant association between CCT and NCT (p < 0.05). However, no significant association was found between CCT and GAT (p > 0.05). This is due to the viscoelastic property of the cornea in which stiffness is associated with the degree of application of tension (20–22). Similar studies have reported that NCT is affected more by CCT than GAT (23,24). In another study among a keratoconus population, the same findings were reported (25). In contrast to these findings, other studies have reported that there is a significant correlation between CCT, NCT and GAT (10–12,14–16).

There was a significant association between CCT and age (p < 0.05). This is similar to studies that have reported that as one ages, central corneal thickness decreases (17,18). Cornea thinning which manifests with aging is initiated through a reduction in the density of keratocytes, collagen fibers degeneration and a decrease in the distance between fibers in the cornea (17,18). Other researchers have reported that the thinning of the cornea can be attributed to the decrease in endothelial density as one ages (19). However, age and gender did not confound the relationship between CCT and NCT and GAT readings of IOP (Table 4).

Gender and ethnicity were found to be independent of CCT. An interracial difference has been observed in the measurement of CCT (17,19). The lack of association between ethnicity and CCT in this study could be explained by the fact that the ethnic grouping among most African populations is based on language and culture. The impact of gender on the measurement of CCT has been studied, and variant views have been presented by researchers. Similar to this study, Lee et al. (26) and Aghaian et al. (27) reported that gender does not affect the measurement of CCT.

In summary, this study found a significant association between CCT and NCT measures. However, there was no significant association between CCT and GAT measures. CCT had an association with age but not with ethnicity and gender. CCT was found to be independent of gender and ethnicity since there was no significant relationship between these variables. This has an implication for intraocular pressure measurements in routine clinical practice. Thus, CCT should be considered in the use of NCT measures of IOP.
